# Integrating spaceborne LiDAR and Sentinel-2 images to estimate forest aboveground biomass in Northern China

**DOI:** 10.1186/s13021-022-00212-y

**Published:** 2022-09-01

**Authors:** Fugen Jiang, Muli Deng, Jie Tang, Liyong Fu, Hua Sun

**Affiliations:** 1 Research Center of Forestry Remote Sensing and Information Engineering, Central South University of Forestry and Technology, Changsha, 410004 China; 2 Key Laboratory of Forestry Remote Sensing Based Big Data and Ecological Security for Hunan Province, Changsha, 410004 Hunan China; 3 Key Laboratory of State Forestry Administration On Forest Resources Management and Monitoring in Southern Area, Changsha, 410004 Hunan China; 4 Research Institute of Forest Resource Information Techniques, Chinese Academy of Forestry, Beijing, 100091 China

**Keywords:** Aboveground biomass, Carbon cycle and management, Remote sensing, ICESat-2, Google earth engine, Machine learning

## Abstract

**Background:**

Fast and accurate forest aboveground biomass (AGB) estimation and mapping is the basic work of forest management and ecosystem dynamic investigation, which is of great significance to evaluate forest quality, resource assessment, and carbon cycle and management. The Ice, Cloud, and Land Elevation Satellite-2 (ICESat-2), as one of the latest launched spaceborne light detection and ranging (LiDAR) sensors, can penetrate the forest canopy and has the potential to obtain accurate forest vertical structure parameters on a large scale. However, the along-track segments of canopy height provided by ICESat-2 cannot be used to obtain comprehensive AGB spatial distribution. To make up for the deficiency of spaceborne LiDAR, the Sentinel-2 images provided by google earth engine (GEE) were used as the medium to integrate with ICESat-2 for continuous AGB mapping in our study. Ensemble learning can summarize the advantages of estimation models and achieve better estimation results. A stacking algorithm consisting of four non-parametric base models which are the backpropagation (BP) neural network, k-nearest neighbor (kNN), support vector machine (SVM), and random forest (RF) was proposed for AGB modeling and estimating in Saihanba forest farm, northern China.

**Results:**

The results show that stacking achieved the best AGB estimation accuracy among the models, with an R^2^ of 0.71 and a root mean square error (RMSE) of 45.67 Mg/ha. The stacking resulted in the lowest estimation error with the decreases of RMSE by 22.6%, 27.7%, 23.4%, and 19.0% compared with those from the BP, kNN, SVM, and RF, respectively.

**Conclusion:**

Compared with using Sentinel-2 alone, the estimation errors of all models have been significantly reduced after adding the LiDAR variables of ICESat-2 in AGB estimation. The research demonstrated that ICESat-2 has the potential to improve the accuracy of AGB estimation and provides a reference for dynamic forest resources management and monitoring.

## Background

As the largest carbon storage in the biosphere, the forest ecosystem is an important part of the terrestrial ecosystem and plays an indispensable role in the global carbon cycle [1, 2]. A timely understanding of the current situation and dynamic change of forest ecosystem is of great significance for human beings to cope with global climate change, study the global carbon cycle, environmental monitoring, and realize human sustainable development. Accurate evaluation of carbon cycle capacity and carbon storage of forest ecosystem is an important link in quantitative analysis of carbon sink [3, 4]. Forest aboveground biomass (AGB) is one of the main components of forest carbon storage because of its large volume, long-term and large-scale impact on carbon balance [5]. As an important index to evaluate forest quality and forest ecosystem service function, AGB can directly measure carbon sequestration capacity [6]. Rapid and accurate acquisition of large-scale AGB is the basic work of forest resource management and ecosystem dynamic monitoring, which is of great significance for studying ecosystem interaction and formulating relevant policies in the process of achieving carbon neutralization [7, 8].

Remote sensing technology has the potential to quickly obtain the growth status of large-scale vegetation, which provides an effective reference for the monitoring and management of forest resources [9]. Extracting vegetation information from remote sensing images and combining it with a small amount of ground-measured data for modeling has become an effective and popular way of obtaining regional AGB [10]. Spectral reflectance can reflect the differences between ground objects, which is the theoretical basis of remote sensing inversion of forest parameters. Optical images are remote sensing data with the widest coverage, the most types and the richest time series in the world [11]. The rich spectral information of optical images can effectively reflect the distribution and growth of vegetation and has been widely used in vegetation classification and forest resources monitoring [12, 13]. Moderate-resolution Imaging Spectroradiometer (MODIS), as a representative of low spatial resolution optical images, has periodic land surface coverage on a large scale, which enables national and even global monitoring of land and vegetation changes. However, the coarse spatial resolution leads to an excessive amount of mixed features in the pixels, which makes the accuracy of identifying ground objects limited [14]. Remote sensing images with high spatial resolution have the potential to recognize surface objects more accurately. However, cloud cover, coverage, and high price lead to limitations in applications over large areas [15]. Due to the moderate spatial resolution, complete coverage and short revisit period, the medium spatial resolution data represented by Sentinel-2 and Landsat is still the most popular and widely used optical remote sensing image. Compared with Landsat data, Sentinel-2 carries more than three red edge bands which are more sensitive to vegetation growth, so it can provide more accurate land change and vegetation growth information [16]. In addition, the time-series data provided by Sentinel-2 makes it possible to obtain high-quality seasonal forest change, which can be effectively used for forest resource monitoring and dynamic management [17, 18]. Google earth engine (GEE) is a cloud-based geospatial processing platform, which can be used for large-scale terrestrial ecosystem monitoring. GEE archives a large number of remote sensing data for public use, and users can directly apply their algorithms to these data [19]. Due to its high efficiency, GEE has been widely used in land cover and land use change (LCLUC) assessment, disaster management, and forest monitoring [20]. GEE has integrated a variety of data including MODIS, Sentinel, Landsat, etc., which can be effectively applied to forest resource monitoring. Utilization of GEE to acquire and process Sentinel-2 data provides the potential to rapidly achieve high-precision forest AGB estimation and mapping on a large scale [21, 22].

Compared with optical remote sensing images, active remote sensing data sources such as synthetic aperture radar (SAR) and light detection and ranging (LiDAR) can penetrate the vegetation canopy to reach the ground surface and obtain information on the vertical structure of the forest stand, thus enabling more accurate estimation of forest parameters [23,24 25]. However, SAR must work in bands with specific frequency, and Gleason et al. [10] found that these bands are usually not necessarily suitable for AGB estimation of all forest types. And because most of the forests are located in the complex terrain area, how eliminating the influence of the elevation fluctuation terrain on the signal is the one of the critical factors limiting the SAR data [23]. In addition, saturation in high AGB areas limits more applications of SAR [24].

LiDAR is another commonly used active remote sensing method, which obtains object features by transmitting and receiving detection signals to the target [25]. Among all LiDAR systems, spaceborne LiDAR, with its high orbit and wide observation area, is the only payload that can rapidly obtain three-dimensional spatial information on large scales or even the global surface [26]. ICESat-2 (Ice, Cloud, and Land Elevation Satellite-2) is one of the latest spaceborne LiDAR systems with a high repetition rate and high sensitivity, which is the first-time applying photon-counting LiDAR (PCL) technology to a satellite platform [27]. ICESat-2 is equipped with an advanced terrain laser altimeter system (ATLAS) using a sensitive single-photon detector. It has a high pulse repetition rate and can obtain a small spot and high-density photon point cloud data, to achieve more accurate three-dimensional surface information traction. ICESat-2 can provide forest vertical structure parameters and effectively alleviate the saturation of optical remote sensing images [28, 29]. Using ICESat-2 to quickly obtain global forest dynamic change information provides the potential to reveal vegetation canopy height, AGB distribution, and change pattern in a large area. However, the space-borne LiDAR data are not spatially continuous, it is necessary to combine optical or other continuous remote sensing data to obtain the continuous spatial distribution of AGB [30, 31].

Parametric and nonparametric methods are commonly used for forest parameter estimation [18]. Parametric models are simple in form and easy to implement, but poor in fitting in complex forest parameter estimation [17]. Nonparametric methods such as backpropagation (BP) neural networks, k-nearest neighbors (kNN), support vector machines (SVM), and random forests (RF) do not require assumptions about sample distribution and can suppress overfitting, and have been shown to be effective for forest AGB estimation [31, 32]. However, due to the complexity of the forest environment, the applicability of these models is inconsistent for different forest types. In addition, the estimation accuracy of these models being used individually is always limited due to factors such as sample distribution, modeling variables, and hyperparameters [32]. Ensemble learning, represented by the stacking algorithm, integrates the advantages of multiple base models and can be effective in achieving higher accuracy forest parameters estimation [31]. However, the effectiveness of stacking constructed with nonparametric models as base models for AGB estimation still needs to be validated. The study aimed to propose a stacking algorithm for AGB estimation and continuous AGB mapping in Northern China. Non-parametric methods including the BP, kNN, SVM, and RF models were used as the base model to construct stacking and to perform the comparison of AGB estimation. The Sentinel-2 images provided by google earth engine (GEE) were used as the medium to synergize with ICESat-2 and measured AGB collected in Saihanba forest farm was used for results validation of the models. In addition, the influence of ICESat-2 variables on the accuracy of AGB estimation was tested and discussed.

## Methods

### Study area

**Fig. 1:**
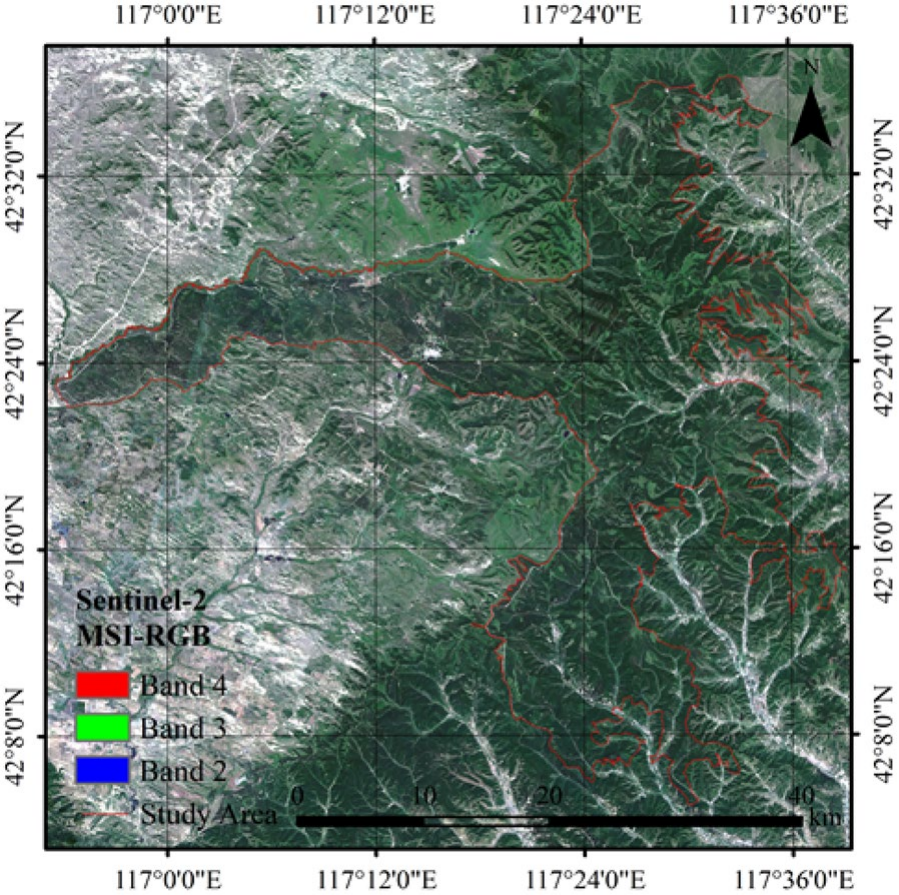
Location and boundary of the study area

This study was conducted in Saihanba Mechanical Forest Plantation, which is the largest forest farm of plantation in China. Saihanba is located in Hebei province, northern China (116°51′–117°39′ E, 42°02′–41°36′ N) (Fig. 1). The altitude of Saihanba ranges from 1010 to 1940 m, with a temperate continental monsoon climate. The annual average temperature, frost-free period, and average annual precipitation in Saihanba are − 1.3 °C, 68 days, and 490 mm, respectively. Larch (*Larix ologensis*), Scots pine (*Pinus sylvestris*), Birch (*Betula platyphylla* Suk), and Spruce (*Picea asperata* Mast) are the dominant tree species. Saihanba forest farm has a forest coverage rate of more than 80% and a total forest stock volume of 10.12 million m^3^, which is one of the main sources of timber provided in China.

### Remote sensing data acquisition and processing

**Fig. 2:**
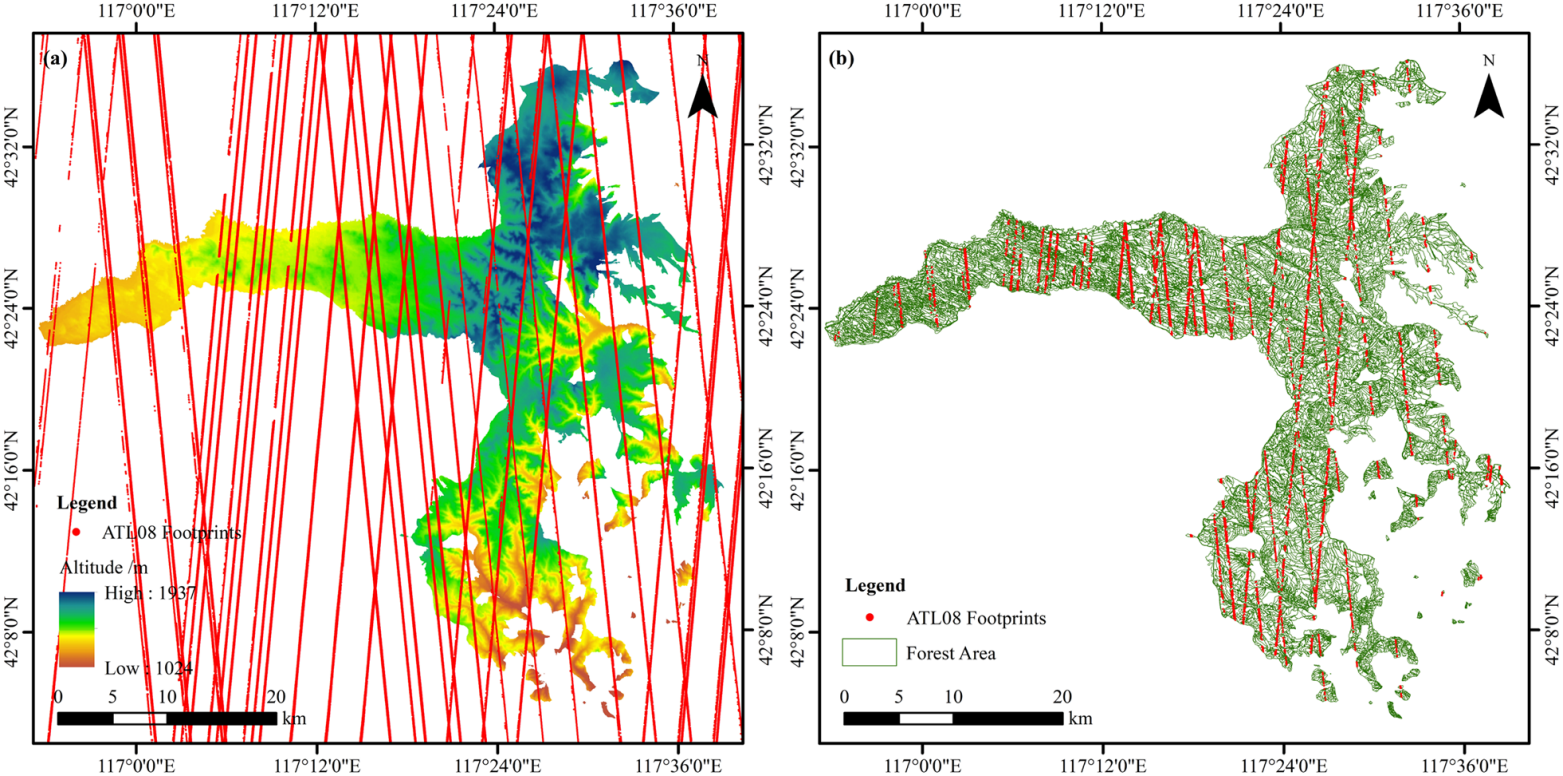
**a** The altitude distribution with the ICESat-2 data and **b** the selected ATL08 segments covering within forest land in Saihanba

ICESat-2 was launched on September 15, 2018, which is equipped with the ATLAS that emits three pairs of beams. The distance between each pair of beams is about 3.3 km, and the distance between the two ground tracks in one beam pair is 90 m [27]. The ATL08 products (Version 3) of ICESat-2 covering the study area from 2018 to 2019 were downloaded from the National Ice and Snow Data Center (NSIDC) (https://nsidc.org/data/ATL08/) (Fig. 2a). The Differential, Regression, and Gaussian Adaptive Nearest Neighbor (DRAGANN) methods have been developed to identify and eliminate noisy photons for ATL08 production [33]. The ATL08 directly provides height estimate segments s with an interval of 100 m along the track. Each segment has a radius of 8.5 m and contains information on center coordinates, altitude, height metrics, and apparent surface reflectance (ASR). Height metrics in ATL08 include minimum, mean, median, maximum, and multiple height profile quantile of the vegetation canopy height [30, 31]. All segments were masked using the boundary of the study area, and the segments marked as valid in ALT08 were selected. In our study, segments with canopy height greater than 50 m and less than 5 m were excluded to ensure the stability and validity of the data. Most trees below 5 m are in a rapid growth age cycle. Finally, a total of 5396 segments were selected as sample plots for AGB estimation in Saihanba (Fig. 2b).

Google earth engine (GEE) platform was used to obtain Sentinel-2 images and preprocess. And images during the growing season in 2017 with cloud cover less than 5% were obtained. To ensure the stability and reliability of the images, median synthesis was performed for all pixels [34]. Sentinel-2 carries more than three red edge bands which are extremely sensitive to the change of vegetation chlorophyll and have been proved to be effective for forest AGB estimation [17, 18]. Band reflectance and vegetation index are the basic variables for AGB estimation, which can effectively represent the growth status and health level of vegetation [10]. Bands with spatial resolution better than 20 m were selected. Vegetation indices (VIs) are obtained by band combinations and calculations, and can be used to quantitatively describe the growth status of vegetation. In addition, the red-edge vegetation indices from the combination of red-edge bands, which are extremely sensitive to vegetation chlorophyll, can accurately reveal the vegetation health. Vegetation indices are closely related to forest AGB and have been shown to be used as variables for forest parameter modeling and estimation [29–31]. Eight vegetation indices including Normalized Difference Vegetation Index (NDVI), Enhanced Vegetation Index (EVI), Red-green vegetation Index (RGVI), Atmospherically Resistant Vegetation Index (ARVI), Red Edge Normalized Difference Vegetation Index (RENDVI), Red Edge Chlorophyll Index (RECI) and Red Edge Simple Ratio (RESR) [30, 31, 35, 36] were also extracted in the study to establish the coupling relationship with ICESat-2 for continuous AGB mapping (Table 1).

**Table 1:** The spectral variables extracted from Sentinel-2 used in this study

Spectral variable	Description
Band reflectance	B2-Blue, B3-Green, B4-Red, B5-Red Edge1, B6-Red Edge2, B7-Red Edge3, B8-NIR, B8A-Red Edge4, B11-SWIR1, B12-SWIR2
Vegetation index	Normalized Difference Vegetation Index (NDVI)
	Enhanced Vegetation Index (EVI)
	Red-Green Vegetation Index (RGVI)
	Atmospherically Resistant Vegetation Index (ARVI)
	Red Edge Normalized Difference Vegetation Index (RENDVI)
	Red Edge Chlorophyll Index (RECI)
	Red Edge Simple Ratio (RESR)

### Statistics of measured AGB values

The measured AGB used in the study was obtained from the forest management inventory (FMI) database in Saihanba. The FMI is conducted every ten years in China with annual supplementary surveys to update the database, which is one of the main ways to capture dynamic changes in forest resources. FMI can provide scientific data for the quality evaluation of forest resources and the formulation of management policies [37]. Irregular subcompartments containing measured forest data are established in the forest resource database for forest resource management and the database is updated every year by auxiliary investigation. The subcompartments mainly include the attributes of tree species, average canopy height, and tree diameter at breast height (DBH). Tree height was measured by laser altimeter and the diameter tape was used to measure the DBH. The average of three measurements was used as the final result. The updated database of Saihanba forest farm in 2017 was obtained, and the forest data were extracted for AGB calculation.

**Fig. 3:**
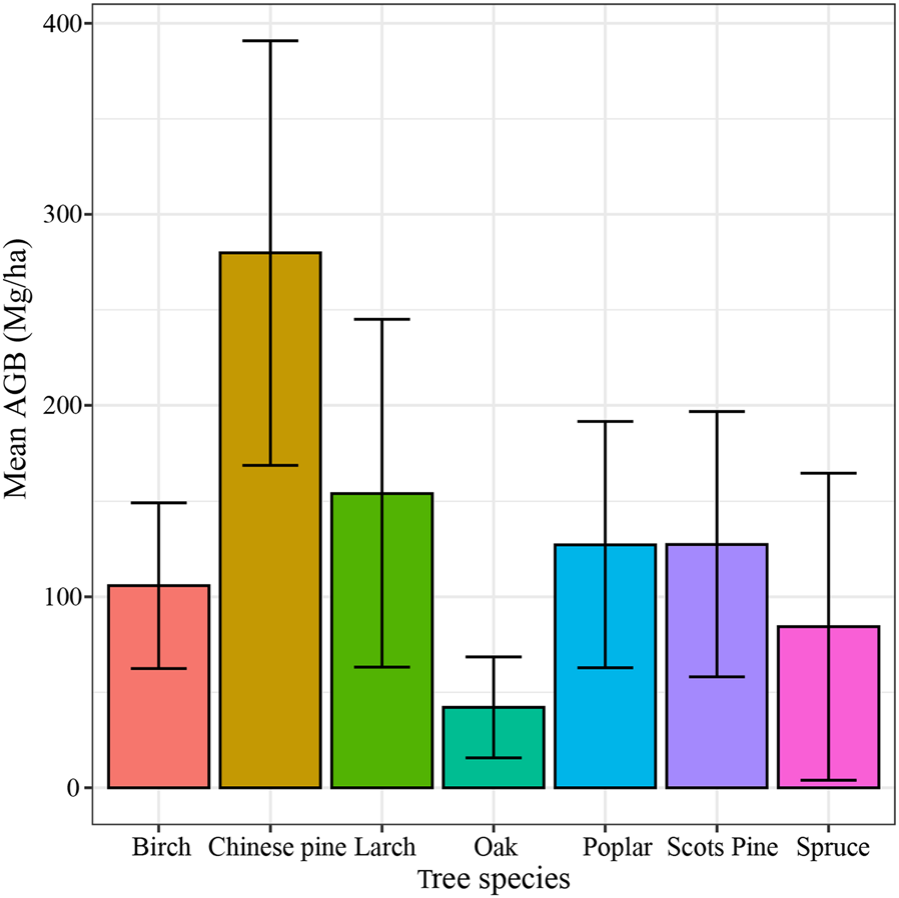
The mean and standard deviation of AGB values under seven tree species in Saihanba

In our study, blocks of FMI covered by ICESat-2 segments were selected for forest attribute extraction. Finally, 5396 sample plots composed of seven tree species were determined in Saihanba (Fig. 2). Larch and Birch are the most widely distributed tree species, separately accounting for 56.1% and 22.4% of the total sample plots. Allometric equations based on different tree species summarized by Li et al. [38] were used to calculate the AGB values of the plots (Table 2). The AGB values of all the samples ranged from 5.14 to 522.68 Mg/ha, and the mean value, standard deviation and coefficient of variation were 135.30 Mg/ha, 82.13 Mg/ha and 60.7% respectively. Among the seven tree species, the mean AGB value of the Chinese Pine was the maximum while the Oak was the minimum (Fig. 3).

**Table 2:** Allometric growth equation based on different tree species for AGB calculation

Tree Specie	Plot number	Allometric equation
Birch	1210	0.0278601(D^2^H)^0.993386^
Larch	3027	0.046238(D^2^H)^0.905002^
Poplar/Oak	218	0.044(D^2^H)^0.9169^ + 0.023(D^2^H)^0.7115^ + 0.0104(D^2^H)^0.9994^ + 0.0188(D^2^H)^0.8024^
Chinese Pine	19	0.027639(D^2^H)^0.9905^ + 0.0091313(D^2^H)^0.982^ + 0.0045755(D^2^H)^0.9894^
Spruce	60	0.067732(D^2^H)^0.865949^
Scots Pine	862	0.3364D^2.0067^ + 0.2983D^1.144^ + 0.2931D^0.8486^
Total	5396	–

### Variable selection

An appropriate variable combination can significantly improve the accuracy of the AGB estimation model. Nonlinear variable selection methods can better describe the relationship between remote sensing variables and AGB under complex conditions compared to linear methods [10]. The importance evaluation based on the random forest algorithm can provide the contribution of all variables to the model, to calculate the relative importance of variables [39]. The main idea is that if the out-of-bag accuracy decreases significantly after adding noise to a feature randomly, it means that this feature has a great impact on the prediction result of the sample, i.e., it indicates that its importance is relatively high [17, 31]. Importance ranking can be effective in screening variables and has been shown to be effective for AGB estimation [40].

To determine the validity of LiDAR variables extracted from ICESat-2 for AGB estimation, the importance ranking, and error change trends were conducted to determine the variable combination of: (1) spectral variables, and (2) spectral variables and LiDAR variables. Finally, the variable combination that obtained the minimum RMSE was used for further modeling. The “randomForest” package [41] in R 4.0.2 was used in the study to calculate the importance of all the feature variables.

### AGB estimation models

The Backpropagation (BP) neural network is a multilayer feedforward neural network trained according to the error backpropagation algorithm. It is one of the most widely used neural network models [42]. However, it is easy to fall into local minimum and result in low learning efficiency, which limits the application of BP in forest parameter estimation. The number of hidden nodes can significantly affect the prediction effect of BP [43]. The range of the parameter was set from 2 to 500 and the number of hidden nodes with minimum RMSE was determined for AGB estimation. The Support vector machine (SVM) can realize regression and classification through a variety of kernel functions, which is sparse and robust. However, when estimating forest parameters, it is complex to determine the specific kernel function and model parameters [31, 44]. And the linear kernel, polynomial kernels, and radial basis function in SVM were constructed to compare the validity of AGB estimation. The kNN uses the k nearest neighbors to represent the attributes of the samples to be tested, which is more suitable for the automatic classification of class domains with large sample sizes [45]. The Mahalanobis distance has been shown to be the most appropriate calculation metric of kNN for vegetation parameter estimation. In addition, the number of nearest neighbors k directly influences the prediction results of kNN [18]. In the study, k was set from 2 to 50, and the k achieving the minimum RMSE was used to determine the final kNN model parameters. Random forest is one of the ensemble algorithms, which constructs a large number of decision trees for prediction [39]. Nonparametric models have been proved to be effective in estimating vegetation parameters. However, for forest ecosystems, the estimation accuracy of these models when used independently is still limited [31]. Random forests have strong noise immunity and can effectively handle high-dimensional data, and have been shown to achieve satisfactory accuracy and robustness for vegetation parameter estimation such as leaf area index (LAI), growing stem volume (GSV), etc., [40]. The mtry and ntrees are the main parameter groups that affect the effect of RF modeling and estimation. mtry represents the bifurcation number of the constructed decision tree, and ntrees is the number of decision trees [39].

**Fig. 4:**
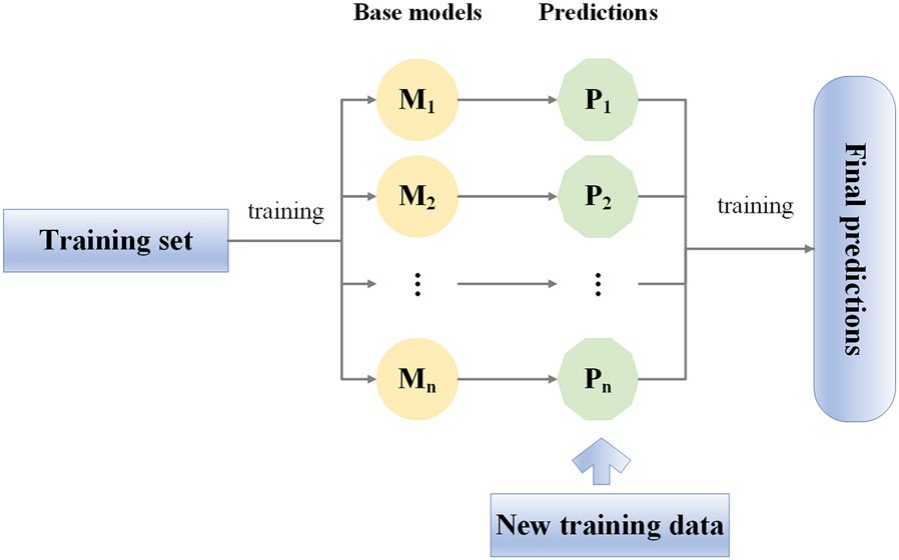
The basic framework of the stacking method

The stacking algorithm accomplishes model training by constructing and combining multiple base models, which often results in significantly superior prediction and generalization performance than a single model. First, stacking uses the base models for training and modeling to obtain predictions; then, the predictions from all base models are integrated as new training samples to obtain new predictions. The stacking can integrate and balance the outputs of all base models, which can effectively improve prediction accuracy and reduce estimation errors. To synthesize the advantages of the nonparametric models and improve the estimation accuracy, a stacking was proposed for AGB estimation in the study. The BP, SVM, kNN, and RF models were regarded as based models to estimate AGB, and then their predicted values were used as new training samples to conduct a new model (Fig. 4). The final prediction values were the prediction result of the stacking algorithm integrated with the base models. All the models were built and calculated in R 4.0.2.

### Accuracy assessment

To verify the estimation effect of the model, seventy percent of the samples were randomly selected as training samples (70%, n = 3777) to build the model, and the remaining thirty percent (30%, n = 1619) were used for validation. The coefficient of determination R^2^ was used to measure the effect of fitting between the predicted and observed values and the root mean square error (RMSE) was used to calculate the estimation error of the models [46]. A larger R^2^ represents the better fitting between the observed value and the predicted value. The smaller the RMSE, the smaller the error of model estimation.

1 $${\mathrm{R}}^{2}=1-\frac{{\sum }_{i=1}^{n}{{(y}_{i}-{\widehat{y}}_{i})}^{2}}{{\sum }_{i=1}^{n}{{(y}_{i}-\overline{y })}^{2}},$$

2 $$\mathrm{RMSE}=\sqrt{\frac{{\sum }_{i=1}^{n}{{(\widehat{y}}_{i}-{y}_{i})}^{2}}{n}},$$

where ${y}_{i}$ is the measured AGB values, $\widehat{{y}_{i}}$ is the estimated AGB values, and n is the sample size.

## Results

### Variable selection and AGB estimation

**Fig. 5:**
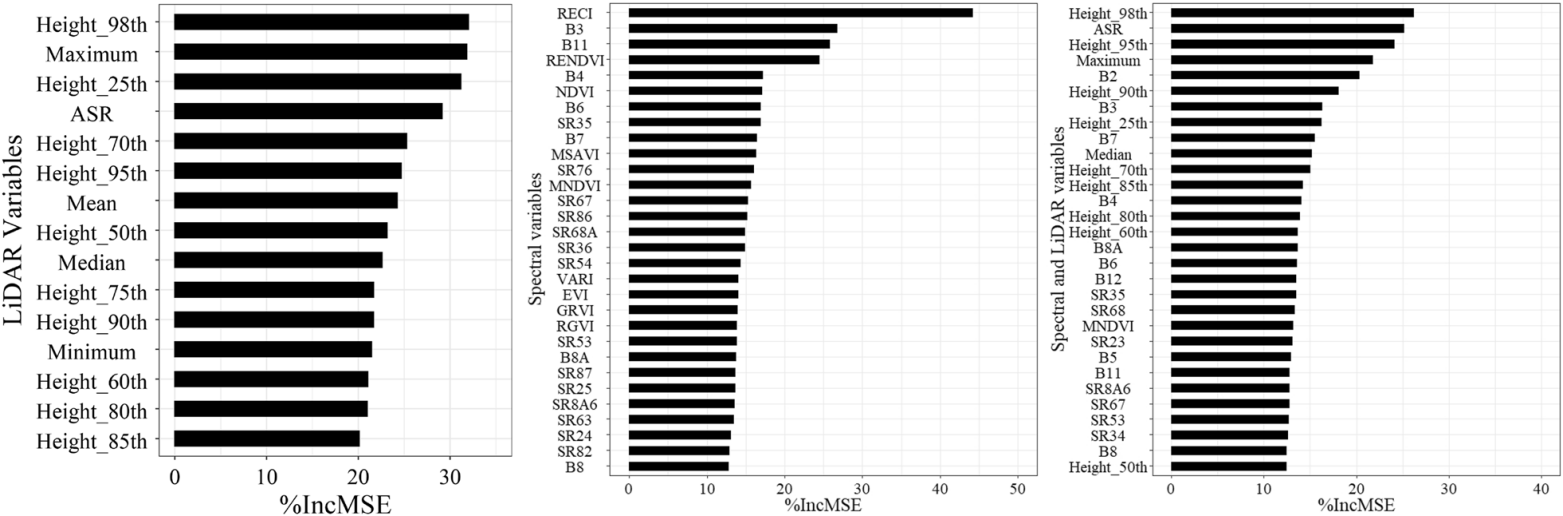
Partial importance ranking of the LiDAR variables extracted from ICESat-2

By ranking the importance of all variables extracted from ICESat-2, 98th percentile height achieved the highest ranking, indicating that 98th percentile height has a significant relationship with AGB. Followed by the maximum and 25th percentile height, however, 85th percentile height got the lowest importance. Figure 5 showed the partial importance ranking of spectral variables and the combination of spectral variables and LiDAR variables of ICESat-2 respectively. In addition, the red-edge vegetation index maintained a relatively high importance among the spectral variables. For the combination of variables in the spectral variables and the LiDAR variables, the LiDAR variables provided a higher importance ranking overall.

**Fig. 6:**
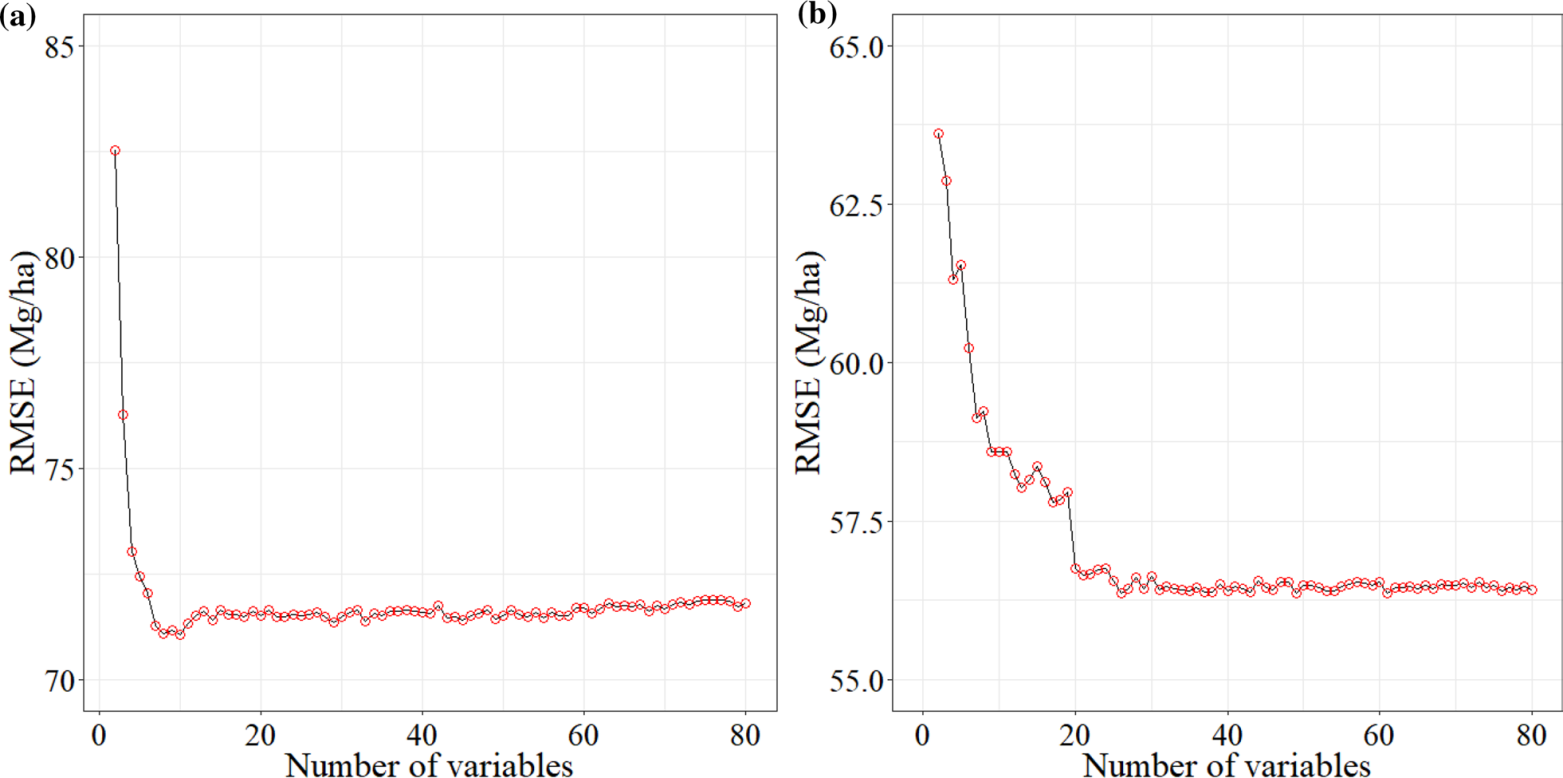
RMSE change based on **a** spectral variables, **b** spectral variables and LiDAR variables

The RMSE of the estimation model based on spectral variables mainly ranged from 70 to 85 Mg/ha. However, after adding the LiDAR variables, RMSE had decreased significantly, ranging from 55 to 65 Mg/ha (Fig. 6). The results showed that when the number of variables were 10 and 26, the RMSE respectively achieved the smallest value and maintained stability.

### Comparison of the AGB estimation results

**Fig. 7:**
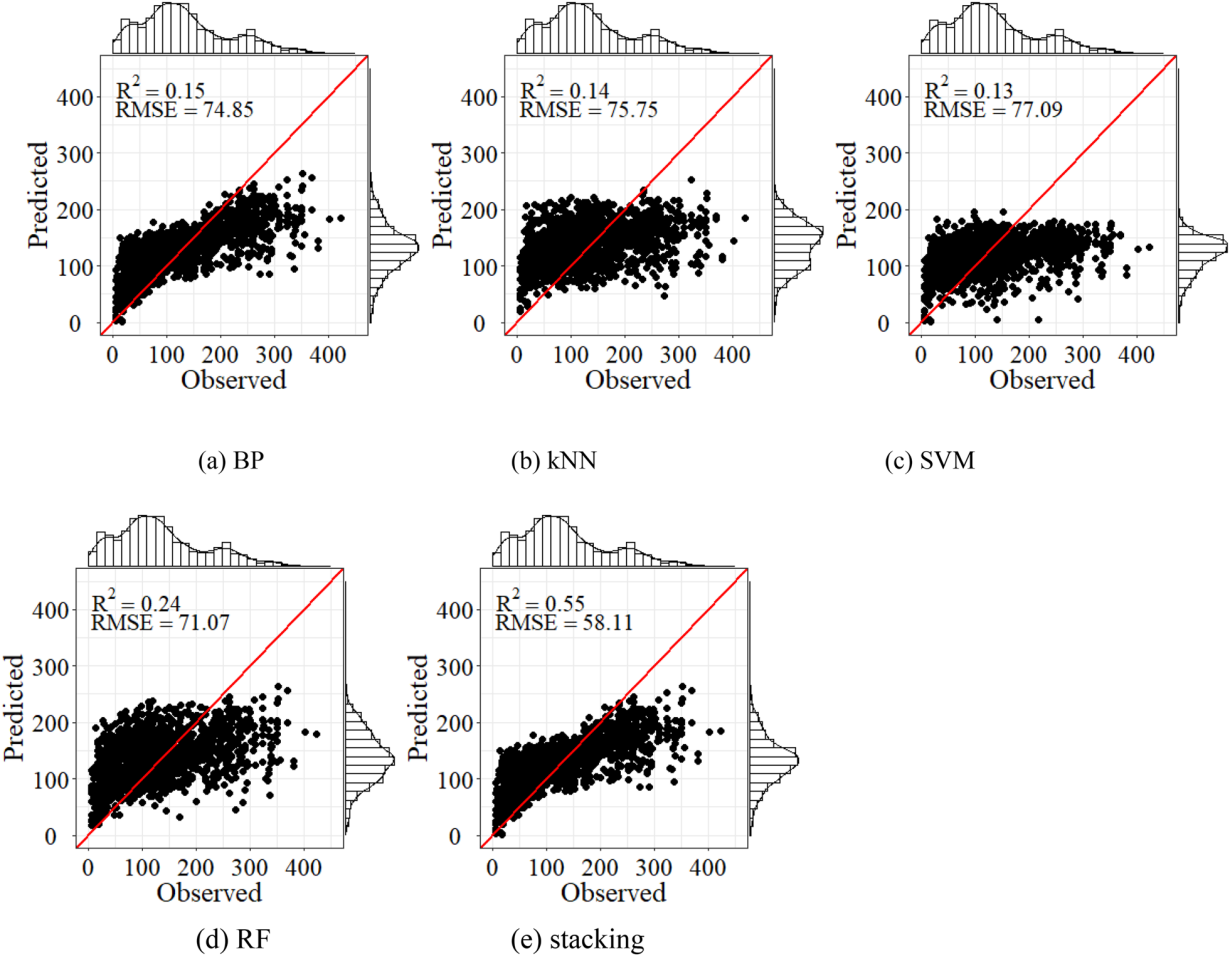
Scatter plots of the observed AGB against the predicted values by **a** BP, **b** kNN, **c** SVM, **d** RF and **e** stacking using the spectral variables (Mg/ha)

**Fig. 8:**
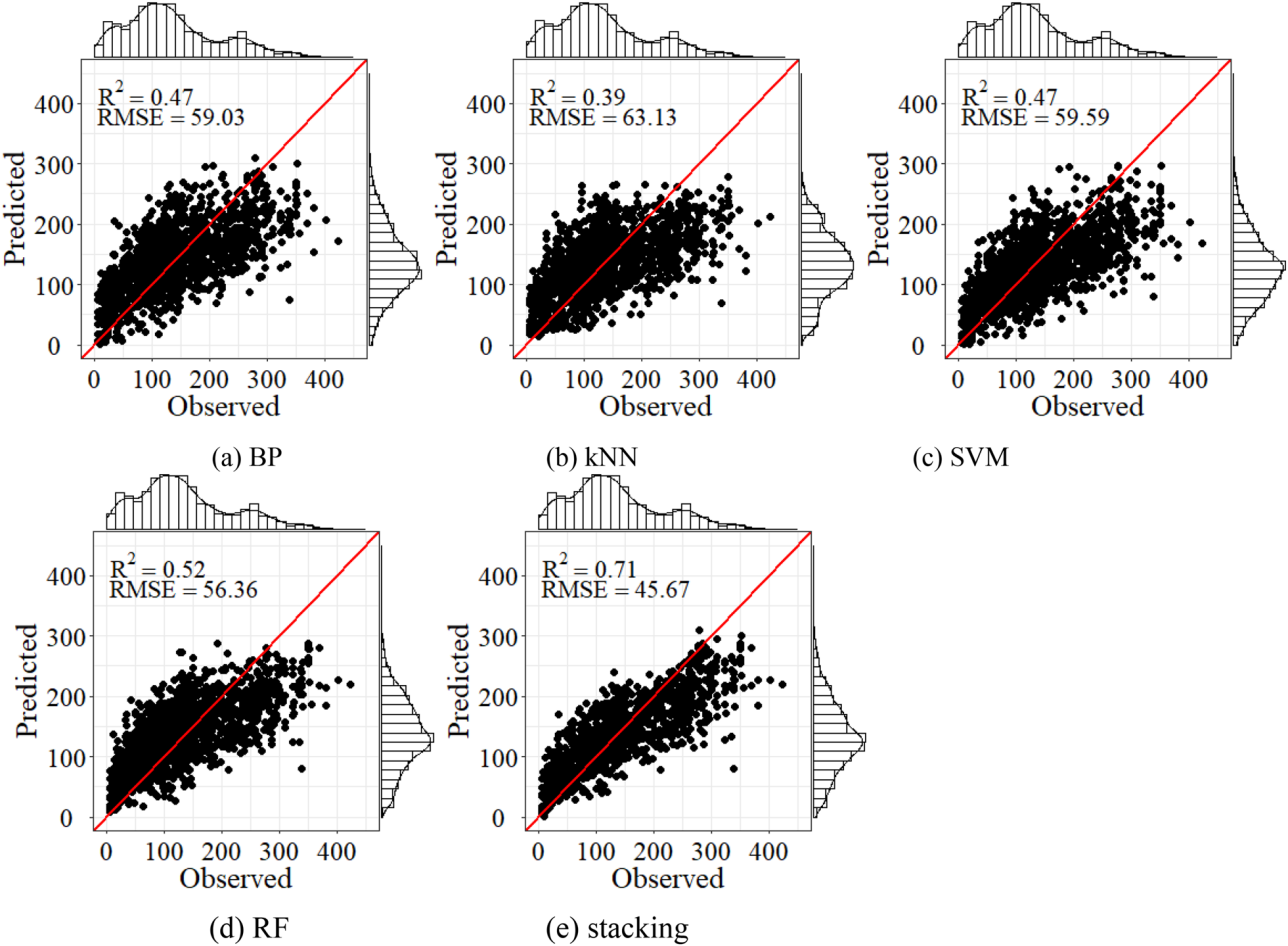
Scatter plots of the observed AGB against the predicted values by **a** BP, **b** kNN, **c** SVM, **d** RF and **e** stacking using the spectral variables and LiDAR variables extracted from ICESat-2 (Mg/ha)

Estimation models were constructed by the variable selection results based on importance evaluation. The BP, SVM, kNN, RF, and stacking models were established for AGB estimation in Saihanba. Figure 7 showed the fitting effect of AGB estimation using Sentinel-2 only. The results of the BP, SVM, kNN, and RF were similar, and the determination coefficients were less than 0.3. However, after combining ICESat-2, the fitting effect of all models has been significantly improved (Fig. 8). Compared with only considering the spectral variables of Sentinel-2, adding the LiDAR variables extracted from ICESat-2 can significantly reduce the AGB estimation error. The stacking always achieved the highest R^2^ and the lowest RMSE. Compared with BP, SVM, kNN, and RF model, RMSE of stacking decreased by 25.7%, 29.3%, 25.1%, and 20.8% respectively when combining ICESat-2.

### Continuous AGB mapping

**Fig. 9:**
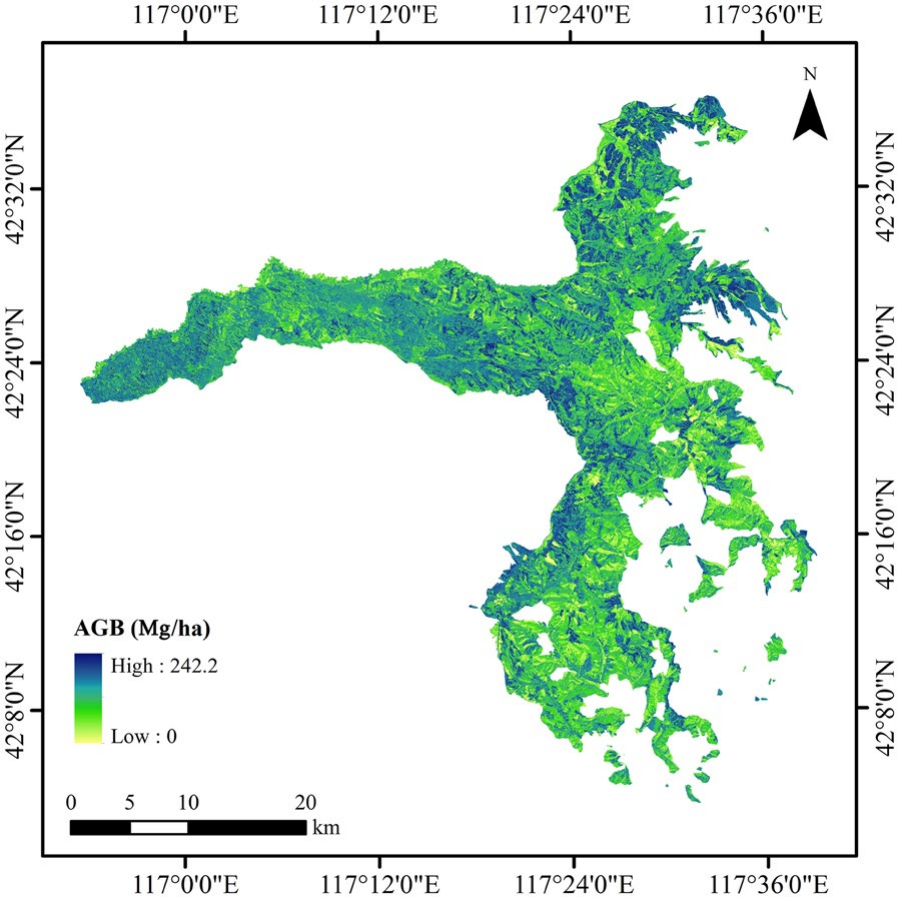
Continuous spatial distribution of AGB in Saihanba

In order to obtain the continuous mapping, the AGB values predicted by the stacking algorithm were taken as the derivative results, and then integrate the continuous Sentinel-2 images for continuous mapping. The spectral variables extracted from Sentinel-2 were calculated for relative importance ranking, and the variable combination used for AGB mapping was determined. Figure 9 showed the continuous spatial distribution of AGB in Saihanba. The smallest AGB predicted values were distributed in the west of the study area, while the larger values were mainly distributed in the northeast and south. And the AGB values in the southeast were lower than that in the West. The predicted spatial pattern of AGB conformed to the actual distribution of Saihanba.

## Discussion

### Variable selection methods for AGB estimation

**Fig. 10:**
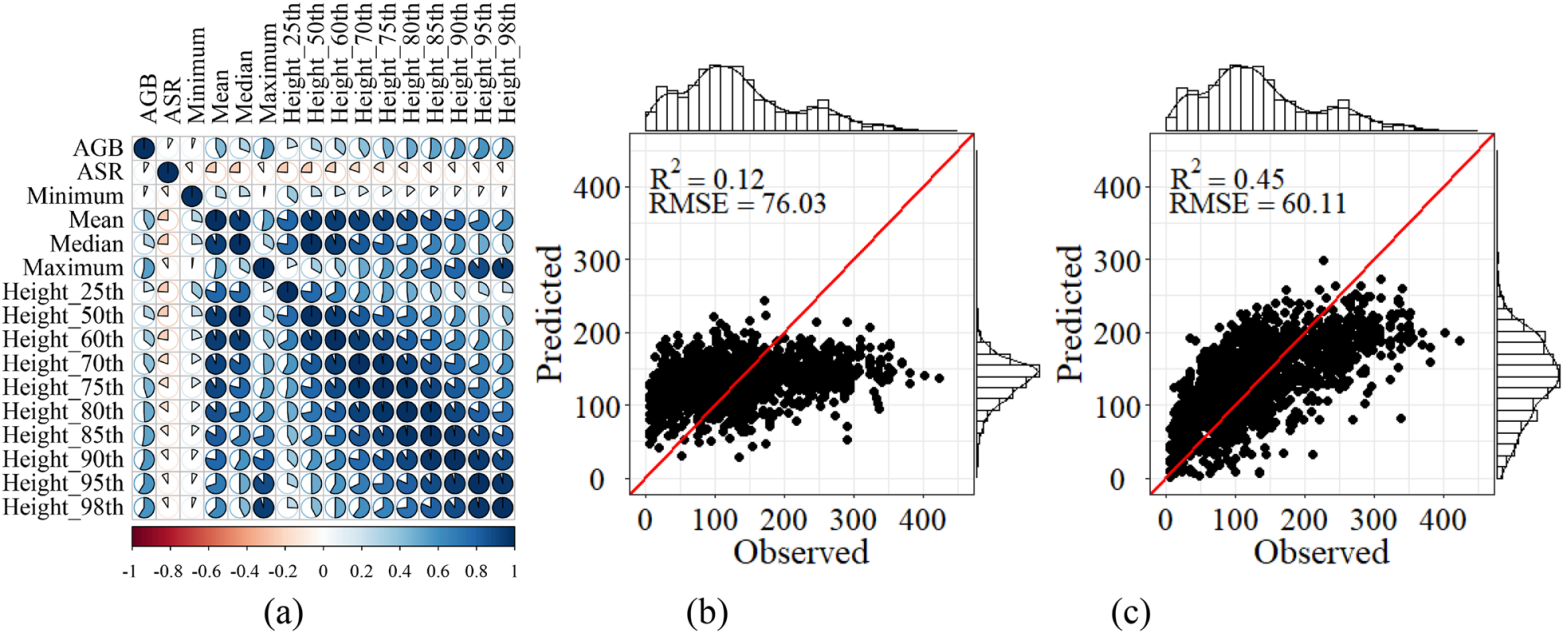
**a** Correlation coefficient matrix of LiDAR variables and AGB, and scatter plots of the observed AGB against the predicted values by linear regression using **b** the spectral variables, and **c** spectral variables and LiDAR variables (Mg/ha)

The combination of variables can directly affect the estimation accuracy and operation efficiency of the model. Linear stepwise regression is one of the most commonly used variable selection methods, which can quickly screen out variables significantly linearly related to AGB [47]. However, due to the complexity and instability of the forest ecosystem, the linear method is limited in AGB estimation [48]. Importance evaluation provides a nonlinear variable selection process with greater potential than the linear method [49]. In order to verify and compare with the variable screening method based on importance evaluation, Pearson correlation coefficient was used to test the linear relationship between all variables and AGB, and the stepwise regression was used to screen the variable combination for establishing the linear model. Figure 10a showed that 98th percentile height achieved the highest correlation with AGB in LiDAR variables and showed a positive correlation of 0.59 (*P* < 0.01). And the RMSE of the linear regression model using spectral variables and the combination of spectral variables and LiDAR variables were 76.03 Mg/ha and 60.11 Mg/ha respectively, which was 23.6% and 24% higher than stacking respectively. In addition, the linear model also showed a significant decrease in RMSE after adding LiDAR variables (Fig. 10).

### Uncertainty, limitations, and prospects

There are many uncertain factors such as remote sensing image types, modeling variables, estimation models that can affect AGB estimation, resulting in uncertainty and estimation error [10, 29, 31]. In our study, Sentinel-2 images were used for modeling and as a medium for continuous AGB mapping. Sentinel-2 carries more than three red bands that are extremely sensitive to forest chlorophyll changes, which is very effective for forest AGB estimation [32]. Due to cloud cover and revisit period, it is difficult to obtain high-quality Sentinel-2 images that are completely consistent with the measured time survey of the sample plot. More importantly, the reflectance obtained by Sentinel-2 in a single period may not accurately reflect the real forest conditions due to sensor errors and the influence of the atmosphere. In the study, the google earth engine (GEE) platform was used to obtain and preprocess Sentinel-2 images. And images during the growing season (from July to September) in 2017 with cloud cover of less than 5% were obtained. To ensure the stability and reliability of the images, median synthesis was performed for all pixels. Optical images can be used to extract band reflectance, vegetation index, and other variables used for AGB modeling and estimation [40]. To reduce invalid information and redundancy, we use the importance evaluation was used in the study to screen variables to improve the accuracy and efficiency of the model. In the study, ICESat-2 was used to extract canopy structure variables. The results showed that the inclusion of LiDAR-derived canopy structure variables can significantly improve the accuracy of AGB estimation model. LiDAR can penetrate the forest canopy to obtain the information of forest vertical structure, which can alleviate the saturation common with using optical imagery [25]. ICESat-2 is one of the latest launched spaceborne LiDAR, which can obtain global vegetation height parameters and has the potential for high-precision and high-efficiency monitoring of large-scale forests [27–30]. However, the segment diameter of 17 m may not be sufficient to accurately describe the ground object information inside the segment. Magruder et al. [50] demonstrated that the average effective diameter in White Sands Missile Range in New Mexico and along a segment of the 88°S line of latitude in Antarctica is about 10–11 m. For forest ecosystems with complex terrain and different vegetation types, the determined and specific effective diameter is very meaningful and needs to be verified. The Global Ecosystem Dynamics Investigation (GEDI) is another spaceborne LiDAR data source that can provide data and services. GEDI provides vertical canopy waveform information between 52°N and 52°S latitudes, which further complements the acquisition method of spaceborne LiDAR data [51]. Compared to ICESat-2, which provides a canopy height product, GEDI can acquire waveform data containing physical properties within the light patch, which allows for more efficient detection of different types of forests. However, the GEDI segments currently covering the same area are limited due to the short duration of satellite launches and revisit period. In addition, similar to ICESat-2, the along-track segments make it difficult to obtain wall-to-wall AGB spatial distribution. Spaceborne LiDAR such as ICESat-2 and GEDI has great potential in estimating global forest height, AGB, and carbon sink [52, 53], but how to synergize optical data and other spatial continuous environmental variables to achieve higher precision continuous mapping is the focus of attention in the future [54, 55].

In complex forest ecosystems, the relationship between remote sensing variables and AGB may not be simple linear, which limits the effect and application of the linear variable selection method [48, 49]. The nonlinear method based on importance evaluation has been proved to be significantly better than the linear method in our study. In order to test the influence of variables on the uncertainty of AGB estimation, the T-test was conducted to examine the relationship between modeling variables and absolute residuals. And the results showed that all variables and residuals were not statistically significant, indicating that variables selected by importance evaluation do not cause significant estimation error and uncertainty.

Parametric models and nonparametric models are commonly used AGB estimation models [10, 18]. The process of parameter model implementation is simple, but it is prone to overfitting and has low stability. Nonparametric models such as machine learning methods have gradually become popular in AGB estimation [17, 56]. However, the application of a single model in the complex forest ecosystem is always limited. Ensemble learning can summarize the advantages of all base models, thereby improving estimation efficiency and accuracy [57, 58]. Even if one base model gets the wrong prediction, other base models can correct the error to achieve better prediction [52]. Jiang et al. [31] demonstrated that it is feasible to construct stacking models for estimating forest canopy height with synergistic data sources of ICESat-2 and Sentinel-2. Generally, forest canopy height is used as an intermediate variable for forest AGB calculation through the allometric equations. In Saihanba, canopy height information extracted from the ATL08 product of ICESat-2 was used directly for AGB estimation, which can reduce the indirect transfer error. Moreover, the nonparametric models were used to construct the stacking, which avoided the overfitting easily caused by parametric models, especially MLR. And the overall accuracy of nonparametric methods was better than MLR in the study, which can effectively improve the accuracy of AGB estimation. Compared with the original base models, the RMSEs of stacking constructed by nonparametric models were reduced by 19% to 27%, which can significantly improve the estimation accuracy. Furthermore, the combination of Sentinel-2 variables and LiDAR variables for AGB estimation was emphasized in the study. Compared with using only spectral variables of Sentinel-2, the RMSE of the joint variables was reduced by 21.4%, which can make more full use of remote sensing information, so as to improve the estimation and mapping of AGB.

In addition, GEE can provide seamless optical data, such as Sentinel-2, Landsat, and MODIS, which are free and publicly available. Combining ICESat-2 with optical data and estimation models has the potential to obtain large-scale forest AGB efficiently [30, 59, 60].

## Conclusions

LiDAR can penetrate forest canopy and obtain more accurate vertical structure information, which has the potential to improve AGB estimation. In this study, optical remote sensing data were used to synergize with spaceborne LiDAR data to realize the large-scale continuous spatial pattern of AGB mapping with high accuracy. ICESat-2 and Sentinel-2 were respectively acquired for extracting LiDAR variables and spectral variables. Nonparametric models and a stacking model were constructed to estimate AGB for comparison and validation. The results showed that the participation of the LiDAR variable can significantly improve the AGB estimation accuracy and reduce the error compared with using spectral variable only. The stacking model achieved the highest AGB estimation accuracy and the lowest RMSE, whose RMSE was reduced by 19% to 27% compared with the base models. In addition, the nonlinear variable selection method based on importance evaluation was proved to be better than the linear method in AGB estimation in Saihanba.

## Data Availability

The data are available upon a reasonable request to the Authors.

## Abbreviations

AGB: Above-ground biomass
ICESat-2: The Ice, Cloud, and Land Elevation Satellite-2
LiDAR: Light detection and ranging
ASR: Apparent surface reflectance
GEE: Google earth engine
SVM: Support vector machine
kNN: K-nearest neighbor
BP: Back propagation
RMSE: Root mean square error
